# The interplay between maternal childhood maltreatment, parental coping strategies as well as endangered parenting behavior during the current SARS-CoV-2 pandemic

**DOI:** 10.1177/25161032211014899

**Published:** 2021-06

**Authors:** Franziska Köhler-Dauner, Vera Clemens, Katherina Hildebrand, Ute Ziegenhain, Jörg M. Fegert

**Affiliations:** 1 27197 University of Ulm, Germany

**Keywords:** Endangered parenting behavior, parental ability for coping strategies, parental childhood maltreatment (CM), SARS-CoV-2-pandemic, transgenerational transmission

## Abstract

The SARS-CoV-2-pandemic is associated different challenges, especially for families. The disruption and challenges require parents to develop strategies to cope with the current situation. One factor that may influence how parents deal with pandemic-associated stressors are experiences of parental childhood maltreatment (CM), which represent a high risk of engaging in endangered parenting. A decisive candidate for the connection between parental CM and the transgenerational transmission could be the parental ability to employ coping strategies. Mothers of a well-documented birth cohort for investigating the pathways leading to resilience or vulnerability in the transgenerational transmission of CM were imbedded in an online “SARS-CoV-2 pandemic survey” assessing maternal ability for coping strategies and the dimension of endangered maternal parenting behavior. 91 mothers completed the online survey. To describe the maternal CM, data from a longitudinal survey were used. Our mediation analysis shows a significant positive relationship between the sum of maternal CM experiences, lack of coping strategies and endangered parenting behavior. This suggests a partial mediation of the association between CM and endangered parenting behavior as the direct effect remained significant when the maternal lack of coping strategies was included as the mediator. Parental CM is a risk factor for coping with stressful situation as well as for endangered parenting behavior. The ability to deal with stress seems to have a significant influence on the context of a possible transgenerational transmission of CM. The results underline the need to consider the unique needs of families with children and to support them as to how to overcome the current crisis.

## Introduction

The current situation caused by the SARS-CoV-2-pandemic is associated with serious challenges and constraints for everyone and effects our social life, the politics, the economy and the media worldwide (economic shutdown, contact restrictions, restriction of public life) (Mairhofer et al., 2020).

Various public health efforts have been made to reduce the rapid transmission of the SARS-CoV-2-virus after it emerged, however these efforts have led to unintended consequences. Families are faced with a number of challenges and measures such as, in particular, recommendations for increasing physical distance, sudden closure of schools and childcare, the loss of community programs and jobs, increasing pressure from recession or unemployment, home schooling, lack of social support from grandparents (Fegert et al., 2020; Halvorsen et al., 2020; John Hopkins University, 2020). As a result, families are more likely to experience increased social isolation, inability to access support and educational services, home office / home schooling reconciliation, and economic difficulties that can exacerbate stress in many households. In fact, social isolation increases susceptibility to stress and can have detrimental effects on mental and physical health (Hawkley & Cacioppo, 2010). In addition, recent research has shown that the parental perceived effects of the SARS-CoV-2-pandemic are associated with increased parenting stress and, thus, an increased risk of harsh parenting (Chung et al., 2020). Therefore, it is important to identify predictors for families who are having difficulty coping with the situation and who, in consequence, may need more support.

One factor that may be of importance on how parents deal with pandemic-associated stressors are experiences of childhood maltreatment (CM). A previous exposure to CM increases the risk of negative consequences on an individual psychological, social and biological level even in less stressful phases than the current pandemic (Felitti et al., 1998; Hager & Runtz, 2012; Norman et al., 2012; Rehkopf et al., 2016). Moreover, CM can significantly affect parenting (Baileyet al., 2012; DiLillo & Damashek, 2003) and increase the risk for harmful parenting behavior including maltreatment (Bailey et al., 2012; Chung et al., 2020; Dixon et al., 2005a, 2005b; DuMont et al., 2007; Savage et al., 2019). It is well known that especially mothers with stressful experiences such as abuse and neglect in their own childhood have an increased risk of passing on their traumatic relationship experiences to their children transgenerationally (Caspi et al., 2002; Madigan et al., 2006). The transmission rate is 7–23% (Berlin et al., 2008; Pears & Capaldi, 2001). Researchers have identified associations between maternal child maltreatment (CM) and maladaptive parenting outcomes such as lower parenting competence, greater parenting stress, role reversal and the use of less parenting styles (Alexander et al., 2000; Fitzgerald et al., 2005; Moehler et al., 2007).

However, not all parents with CM experiencing stress from the SARS-CoV-2 pandemic may be at risk of poor parenting, suggesting that protective factors may mitigate the impact of the pandemic on parental behavior. A decisive candidate in the connection between parental CM and the transgenerational transference of these experiences to the next generation in the context of the current health situation could be the parental ability for coping (Dijkstra & Homan, 2016, Walsh et al., 2010).

Specifically, adaptive coping strategies and supportive family environments may serve as protective factors for families experiencing stress and may differentially influence abuse potential (Dijkstra & Homan, 2016).

Research clearly showed that CM can have an impact on how people deal with stressful situations, and coping strategies can, in turn, increase, prolong, or improve the stress response (Sheffler et al., 2019). Despite the definitional differences in its conceptualization, coping can generally be understood as a response to a stressful situation with the aim of psychosocial adjustment (McCrae, 1984). Coping can consist of cognitive or behavioral responses to adversity such as a stressor or stressful situation (Holohan & Moos, 1987). Cognitive coping refers to the skills or strategies utilized to change one’s perception or appraisal of a situation whereas behavioral coping targets actions to reduce the resulting effects of stressors, such as arising distress. These cognitive or behavioral strategies can further be divided into engagement and disengagement coping according to Carver and Connor-Smith (2010). Dijkastra and Homan (2016) argued that engaging coping strategies such as confronting rather than diverting from stressors or their effects led to higher perceived control. In contrast, disengaging strategies increased the experience of lack of control and were found to be related to deteriorated psychological well-being (Dijkastra & Homan, 2016).

Finkelhor and Browne (1985) have considered the influence of experiences of child sexual abuse (CSA) as a form of CM on the development of particular coping strategies in their model. Finkelhor (1987) further suggested that individuals may adopt abuse-directed coping strategies that do not work adaptively in the long-term across a range of different stressful situations. Therefore, the link between CM experiences and coping skills has to be taken into account when investigating their role as risk factors for a susceptibility to stress and poor parenting (Finkelhor, 1987). Accordingly, a perceived lack of control can be derived as one candidate accountable for the effectiveness of coping strategies. Himelein and McElrath (1996) indicated that increased perceptions of control were predictive of better adjustment in individuals with and without a history of CSA. In line with that, coping strategies could function protectively against the effect of CM on vulnerability to stress.

In light of previous research connecting coping strategies with adult adjustment and functioning (Folkman & Lazarus, 1980), it is reasonable to hypothesize that the scope of coping strategies could be affected by a history of CM and partially account for the influence of CM on parenting behavior.

In sum, CM can directly affect the regulation of emotions and the types of coping strategies people use, potentially leading to an even greater susceptibility to physiological and emotional stress. As experiencing CM during childhood can interfere with successful management of stressful situations (Duffy et al., 2018; Hein & Monk, 2017), a significant influence of parental CM via parental ability for coping on the quality of parental behavior during a pandemic can be assumed.

Therefore, the aim of our study is to analyze the interplay between maternal CM, parental coping of the current pandemic as well as harmful parenting behavior.

## Methods

### Study design

The data was collected within the interdisciplinary study TransGen, investigating risk and resilience factors in the transgenerational transmission of childhood maltreatment (CM) in a prospective approach by focusing on psychological, biological and social factors. The study was approved by the Ethics Committee of Ulm University, performed in accordance with relevant guidelines and regulations and funded by the Federal Ministry of Education and Research (October 2013–March 2017) ([blinded)].

Immediately after parturition, women were recruited at the maternity unit of the Ulm University Hospital and screened for CM using the German version of the Childhood Trauma Questionnaire (CTQ) (Bader et al., 2009; Bernstein et al., 2003). Following the recruitment, all mother-child dyads were followed up three times: 3 months (t1), 12 months (t2) after birth and at age 3.

To get an impression in how far the individual scope of coping skills and the quality of parenting behavior was affected by the current SARS-CoV-2-pandemic we asked all participating mothers to take part in an online “SARS-CoV-2-pandemic survey” which was available from May 18th–July 31st, 2020.

### Participants

In total, 533 mother-child-dyads were recruited in the women’s hospital of the University Hospital of Ulm within 1 to 6 days after parturition. Inclusion criteria for sample selection were: age > 18, more than 37 weeks of pregnancy, sufficient knowledge of the German language, no complications during parturition or health problems of mother and/or child as well as no current drug consumption or history of psychotic disorders or current infections. At least 240 mothers provided written informed consent and mother and child could be invited for a follow-up 3 months (t1) after birth (laboratory and home visit). 158 mother-child-dyads participated in a further laboratory and home visit around 12 months of child’s age (t2).

To assess the current burden of the families due to the pandemic all 158 mother-child-dyads were asked to fill in the online “SARS-CoV-2-pandemic survey.” 92 mothers completed the online survey until the end of July 2020. For our analysis, we only considered complete data sets of mother–child-dyads. The number of data assessed is n = 91 because of missing values for one case.

### Measures

The “SARS-CoV-2-pandemic survey” recorded socio-demographic data of the mothers and participating families, respectively, such as the age of the mother, the educational level, the occupation, the marital status, number of persons under 18 years living in the household and the number of own children. In addition, it was recorded whether the mother and her potential partner were currently working in a systemically relevant area and whether the household’s income had decreased by more than a quarter or the workload had changed since the beginning of the pandemic.

The maternal CM experiences were assessed using the German version of the Childhood Trauma Questionnaire (CTQ), a standard tool for retrospective assessment of CM with satisfactory internal consistency immediately after parturition (Wingenfeld et al., 2010). CM is assessed with five items each rated on a 5-point Likert scale. The CTQ subscale scores range from 5 to 25 and the total scores from 25 to 125. The cumulative measure from “none” maltreatment experiences (25 points) over “minimal” to “extreme” maltreatment load (125 points) calculated the sum score of all 25 items (Bernstein & Fink, 1998).

The degree of coping strategies was assessed using the Pearlin Mastery Scale (Pearlin & Schooler, 1978) which assessed an individual regards of chances as being under personal control. The questionnaire depicts the individual scope of coping skills based on 7 items on a 4-point Likert scale (1 = “strongly disagree” to 4 = “strongly agree”). The total scores range from 7 to 28 points. Because of the negative phrasing of items and coding of answers, higher scores indicate a greater inability to exert, and thereby the tendency to have a lack of, individual coping strategies. Therefore, our operationalization of coping strategies is related to the varying degree of perceived control that reflects the individual’s inability to effectively cope or employ adequate strategies to deal with adversity such as stressful situations.

The extent of endangered parenting behavior since the beginning of the pandemic was recorded with the help of 4 items: “I’ve been yelling at the child more,” “I am more impatient with the child,” “Everyday life with the child is very chaotic,” “I am more afraid that my hand will slip against the child.” The items were rated on a 7-point Likert scale (1= “strongly disagree” to 7= “applies very much”). The total score of the items shows the extent of harmful parental behavior during the pandemic so that the higher the score is, the more pronounced the harmful parenting behavior was rated.

In our model we control for the mothers’ age, level of education and changes in income. Mothers’ level of education was assessed by the question “What is your highest level of education?”. To answer the question, the mothers could choose from 4 different answer options to classify their level of education. Changes in income were assessed with the question “Has the income available in your household fallen by more than a quarter since the beginning of the crisis?”. Changes in income were gathered by a binarily coded answer.

### Statistical analyses

Statistical analyzes were conducted in IBM SPSS Statistics Version 27 for macOS. Descriptive statistics were produced to examine the variables’ distributions. Data were assessed for normality graphically with normal probability plots and statistically with the Kolmogorov-Smirnov test. The normal probability plots and the results of the Kolmogorov-Smirnov-test revealed that none of the variables were normally distributed. However, according to Hayes (2017), the procedure we employed for mediation analysis can be considered robust against violations of normal distribution as a statistical requirement.

Homoscedasticity of residuals and linearity were graphically assessed with residual scatterplots, which indicated some heteroscedasticity but no conspicuous problems for linearity. A heteroscedasticity consistent standard error estimator was applied.

Multicollinearity was tested by inspecting the variance inflation factor and tolerance (VIF = 1.134, tolerance 0.882), which were located within the limits acceptable for regression analysis.

The Durbin Watson test revealed no autocorrelation based on the value of 2.028.

We computed Pearson correlations between control and model variables in order to review their bivariate associations before conducting the mediation analysis.

The PROCESS macro in version 3.5 based on the method of ordinary least squares (OSL) for multiple linear regression analysis (Hayes, 2017) was used to test the proposed mediation with the model for simple mediation (model 4). Bootstrapped (n = 10.000) and bias-corrected confidence intervals were computed for the inferential test of the total, direct and indirect effects.

The power of the mediation analysis was conducted based on an application developed and described by Schoemann et al. (2017). Given our sample size of n = 91, the Monte Carlo Power Analysis for Indirect Effects produced a sufficiently high power of 0.88.

## Results

### Descriptive analyzes

The average age of the mothers at the time of the “SARS-CoV-2-pandemic survey” was 38 (M = 38.14 years, SD = 4.08 years) and the children’s age was between 4.98 and 7.14 years (M = 6.03 years, SD 0.61 years). The majority were of German origin (89.6%) and reported to be married or in a partnership (79.1%). Moreover, the mothers had achieved a general high education exemption: grammar school degree (72.8%,); secondary school degree (20.7%); basic secondary school degree (5.4%); no school diploma (1.1%). Most mothers (60.9%) had completed a degree of higher education and only few participants had attended school for less than 12 years (38.9%). Only 6.6% of participants had indicated a decrease in income. All descriptive statistics are presented in Table 1.

**Table 1.** Descriptive statistics of control and model variables.

**Variable**	**M**	**SEM**	**SD**	**Median**	**Min**	**Max**	**Skew-ness**	**Kurtosis**	**%**
**Age children**	6.03		0.61		4.98	7.14			
**Age of mother**	38.14	0.43	4.08	38.00	31.00	46.00	0.12	−0.69	
**German citizenship**	—	—	—	—	—	—	—	—	89.6
**Partnership status**	—	—	—	—	—	—	—	—	79.1
**University degree**	—	—	—	—	—	—	—	—	60.9
**Grammar school degree**	—	—	—	—	—	—	—	—	15.2
**Basic secondary school degree**	—	—	—	—	—	—	—	—	17.2
**No secondary school degree**	—	—	—	—	—	—	—	—	6.5
**No decrease in income**	—	—	—	—	—	—	—	—	92.4
**Decrease in income**	—	—	—	—	—	—	—	—	7.6
**Sum of CM**	6.63	0.30	2.88	5.00	5.00	17.00	2.25	4.45	
**Lack of coping strategies**	13.37	0.39	3.72	13.00	8.00	24.00	0.59	−0.50	
**Endangered parenting behavior**	12.04	0.58	5.50	11.00	4.00	28.00	0.43	−0.31	

The inspection of the histogram and descriptive data of the sum of CM revealed a strong right skew. The median shows that at least half of all participants indicated a low degree of CM. The average sum of CM (M = 6.63) on a range between 5 and 17 underlines this orientation of the distribution with only few extreme values. Analysis of the corresponding boxplot pointed toward nine outliers of which four were located 1.5 times the interquartile range above Q3 and five were located 3 times the interquartile range above Q3. They were not excluded as they were essential for variability in the sample.

Analysis of the histogram for the extent of coping strategies and endangered parenting behavior showed much variability in the data. The mean of the inability to use coping strategies was 13.37 (SD = 3.72) and the median 13, which illustrates that at least half of participants had indicated to have a more severe lack of coping strategies than average.

Endangered parenting behavior (M = 12.04, SD = 5.50) graphically and descriptively revealed that a considerable number of values were located at the end of the lower range, but the distribution was not noteworthily right skewed.

### Coping strategies mediates the interplay between CM and endangered parenting behavior

We computed Pearson correlations between control and model variables in order to review their bivariate associations before conducting the mediation analysis. The results showed that none of the control variables were significantly related to the hypothesized predictor, mediator and criterion of the mediation model. The zero-order correlations between the sum of CM, coping strategies and endangered parenting behavior, however, were found to be highly significant (see Table 2).

**Table 2.** Pearson correlations/zero-order bivariate correlations between variables.

Variable	1	2	3	4	5	6
1. Age of mother	1					
2. Education of mother	0.24*	1				
3. Decrease in income	0.08	0.03	1			
4. Sum of CM	0.20	−0.15	0.08	1		
5. Coping strategies	0.12	−0.09	−0.01	0.34**	1	
6. Endangered parenting behavior	0.01	−0.18	−0.08	0.40**	0.43**	1

*p < 0.05.

**p < 0.01.

Listwise exclusion.

n = 91.

Endangered parenting behavior showed a moderate to strong correlation with the sum of CM, r(89) = 0.40, *p* < 0.001 and lack of coping strategies, r(89) = 0.43, *p* < 0.001. Lack of coping strategies also was found to moderately correlate with the sum of CM, r(89) = 0.34, *p* < 0.001. Therefore, a significant influence between the model variables could be derived and we proceeded with the mediation analysis.

**Figure 1.**
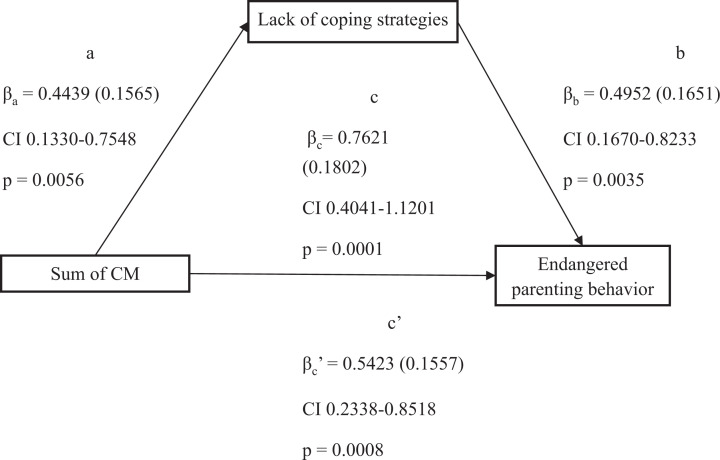
Coping strategies mediates the interplay between CM and endangered parenting behavior.

The total (*p* = 0.0001), direct (*p* = 0.0008) and indirect (95% bootstrap CI 0.0504–0.4456) effects were significant. Unstandardized coefficients with corresponding standard errors, confidence intervals and *p* values as results of the mediation analysis are presented in Figure 1.

As indicated by the bivariate correlations, the sum of CM showed a significant association with lack of coping strategies (β_a_ = 0.4439, SE 0.1565, *p* = 0.0056,). Therefore, mothers with higher sums of CM were more likely to have fewer coping strategies or lack the ability to employ them adequately. Lack of coping strategies further significantly predicted endangered parenting behavior, when controlling for the sum of CM (β_b_ = 0.4952, SE = 0.1651, *p* = 0.0035).

Lack of coping strategies was observed to mediate the relation between the sum of CM and endangered parenting behavior significantly as the bootstrap confidence interval did not include zero (indirect effect: β_a__∗__b_ = 0.2198, bootstrap SE 0.1037, bootstrap CI 0.0504–0.4456). However, under control of the mediator’s influence on the criterion, the direct influence of the sum of CM on endangered parenting behavior remained significant with an effect of β_c_ = 0.5432 (SE 0.1557, CI 0.2328–0.8518). The total effect of the sum of CM on endangered parenting behavior was β = 0.7621 (SE 0.1802, CI 0.4041–1.1201). The results suggest that direct effect is reduced by the significant role of the mediator compared to the total effect. This is consistent with a partial mediation of the association between the sum of CM and endangered parenting behavior as the direct effect remained significant when including lack of coping strategies as mediator.

The model including a direct and indirect effect could explain 27.8% of variance (R^2^ = .278, F(89) = 18.02, *p* < 0.0001) whereas the model that did not include lack of coping strategies as a mediator for the association between sum of CM and endangered parenting behavior could only explain 15.9% of variance (R^2^ = 0.159, F(89) = 17.89, *p* < 0.0001)

When controlling for age of mother, education of mother and decrease in income, the total, direct and indirect effects remained significant and no significant influence of these could be detected (see Figure 1).

## Discussion

The presented study examines the effect of parental experiences of CM on the ability to develop coping strategies and the risk of performing endangered parenting behavior in the focus of the SARS-CoV-2 pandemic. The results showed a significant positive relationship between parental CM, the lack of coping strategies and endangered parenting behavior. The parental lack of coping strategies could be identified as an essential mediator with regard to the connection between the parental CM and the quality of parental behavior.

To the best of our knowledge, this is the first study assessing the interplay of parental CM, coping with a crisis like the SARS-CoV-2 pandemic and parental behavior. It was previously suspected that participants who had experiences of CM were at higher risk for greater sensitivity to stress and threats as well as lower coping skills (Duffy et al., 2018; Fegert et al., 2020; Hein & Monk, 2017). Numerous current studies investigating the pandemic-related stress on parents have shown that the current pandemic leads to high levels of perceived parenting stress (Brown et al., 2020) and parents who have experienced CM are known to have a higher stress-vulnerability (Duffy et al., 2018; Hein & Monk, 2017).

Moreover CM can significantly affect parenting (Bailey et al., 2012) and increase the risk for harmful parenting behavior including maltreatment (Bailey et al., 2012; Dixon et al., 2005a, 2005b; DuMont et al., 2007). It is well known that especially mothers with stressful experiences such as abuse and neglect in their own childhood have an increased risk of passing on their traumatic relationship experiences to their children transgenerationally (transmission rate: between 7–23%) (Berlin et al., 2008; Caspi et al., 2002; Madigan et al., 2006; Pears & Capaldi, 2001).

Current studies on the pandemic-related burden of parenting have shown that regardless of the parent’s relationship-related history, the effects of SARS-CoV-2 pandemic as perceived by the parents are associated with increased parenting stress and, thus, an increased risk of harsh parenting behavior (Chung et al., 2020).

Of course, it should be added that a pandemic, occurring to the extent and intensity of the current one, harbors an attributable risk for burdens. Nonetheless, the studies that are currently available examining the family burden caused by the pandemic show that the extent of stress within young families seems to be unexpectedly high on many levels and that the total and long-lasting extent of the additional stress may not yet be foreseeable (Fegert et al., 2020; Halvorsen et al., 2020; John Hopkins University, 2020).

The results of our investigation complement the results already available in that they show that mothers with CM are at higher risk for having difficulties with coping with the current pandemic. The experience of CM during childhood is associated with a greater level of lack of individual coping strategies (r(89) = 0.34, *p* < 0.001). This is in line with Duffy and colleagues (2018) as well as Hein and Monk (2017) who emphasized the association between CM and the heightened response to threat and decreased coping-mechanisms (Duffy et al., 2018; Hein & Monk, 2017). This can also be seen as consistent with Milojevich et al. (2018) who reported that maltreated adolescents indicated a tendency to have difficulties with coping by drawing on more maladaptive strategies to deal with negative emotions compared to comparison adolescents.

Our analyzes also confirm previous research findings that parental CM poses a risk with regard to performing endangered parenting behavior (r(89) = 0.40, *p* < 0.001).

Maternal experiences of CM during childhood predicted a greater absence of individual coping strategies (*p* = 0.0056) and was not only linked directly to endangered parenting behavior but also indirectly (bootstrap CI 0.0504–0.4456). Lack of coping strategies was found to partially mediate the interplay between CM and endangered parenting behavior. Mothers with more experiences of maltreatment as a child were more likely to have a more severe lack of coping strategies, which also increased the risk for endangered parenting behavior (*p* = 0.0035).

Therefore, a lack of coping strategies should not be neglected when investigating the influence of CM on maladaptive parenting behavior as this effect might be smaller if not for a lack of coping strategies. Nguyen-Feng et al. (2017) reported a significant mediation of the association between childhood emotional abuse and distress through daily avoidant coping. Our study considered CM as a vulnerability factor for stress such as posed by the parenting challenges of the SARS-CoV-2-pandemic. Accordingly, the additional inability for adaptive coping or lack of coping strategies in stressful situations seems to be an essential candidate with regard to the risk of possibly passing on previous adverse relationship experiences.

However, these results also implicate the potential of adopting coping strategies for successfully navigating situations of additional perceived stress such as the parental challenges posed by the SARS-CoV-2 pandemic. Sheffler and colleagues (2019) already pointed out the importance of coping strategies in prevention or intervention approaches for individuals who have experienced CM. In their longitudinal study, less adaptive coping at Wave II partially mediated the relationship between CM at Wave I and psychiatric and physical health outcomes at Wave III. Therefore, the ability to employ coping strategies could function as a compensator or “cushion” for the risk of maternal experiences of CM affecting parenting behavior under such conditions (Sheffler et al., 2019). As Meng et al (2018) concluded in their review, which included studies examining coping as an aspect of resilience, that providing protective factors could prevent or moderate negative consequences following CM. Coping strategies could decrease the risk of endangered parenting behavior caused by an inference from CM experiences (Meng et al., 2018). This could be a starting point for future research on how to help mothers with experiences of CM establish and practice a number of coping strategies in order to attenuate the additional perceived stress of the ongoing pandemic-related challenges that can lead to endangered parenting behavior in the face of maternal CM experiences.

The impairment of parental coping strategies to deal with the current pandemic-associated stressors should not be overlooked when considering how families can be efficiently supported in crises like the SARS-CoV-2 pandemic (Wu et al., 2020). In order to better address the challenges and restrictions posed by a pandemic to family situations and to better protect children’s well-being during a pandemic, affected families should be supported in the context of prevention or intervention approaches with a focus on the support and expansion of parental coping strategies as protective factors for families experiencing stress.

Nevertheless, several limitations of our data have to be considered. First, our sample is restricted to participants from an online survey, which is why the findings cannot be considered representative for the general public and therefore not generalizable for all families. This limitation is also related to the inherent characteristics of our sample as a large number of participants were highly educated, had a partner and did not experience an increase in income due to the pandemic, which points toward the possibility of an attenuated effect size. In their review, Meng and colleagues (2018) found higher education and socioeconomic status to be positively linked with psychological well-being and life satisfaction and considered them protective factors among people with a history of CM. Ford (2012) reported that a lower educational level of women with victimization history was associated with higher severity of trauma-related symptoms in their sample and that action coping was only positively associated with trauma-related symptoms for less educated women. He concludes that posttraumatic resilience may be enhanced through educational attainment that facilitates the access to adaptive coping strategies (Ford, 2012). Therefore, as these characteristics may protect against or reduce the consequences of CM, its effect on harmful parenting behavior through a lack of available coping strategies can be expected to be more pronounced in a more representative sample.

It must be mentioned here that other events within a family can similarly increase the stress for parents in the face of a history of abuse and neglect, unrelated to the specific burdens of a pandemic. Nonetheless, having difficulties coping with the perceived rising stress likely affects the quality of parental behavior as has been observed in the case of the current SARS-CoV-2 pandemic as a source of parenting challenges. This study contributes to the evidence that parents with experiences of CM are at higher risk for struggling with coping and performing endangered parenting behavior in stressful times such as the pandemic. In addition, further analyzes should focus on a potential increase in maternal stress during the pandemic compared to prior the prior level. The study we presented here exclusively focuses on the perceived stress levels of mothers during the pandemic.

Furthermore, due to the short time period for data collection at the beginning of the pandemic we focus on a small sample. In this regard, further studies must be considered to consolidate and expand the model to verify the results focusing on a larger sample size. Questions on parental CM, coping strategies as well as parental behavior are based on retrospective self-report which may impair validity due to social desirability.

The presented results, however, give an important insight into the relevance of factors such as parental CM, coping strategies as well as maladaptive parenting behavior in the focus of a crisis as represented by the current SARS-CoV-2 pandemic.

## Conclusion

Findings highlighted that parental CM marks a risk factor for coping with stressful situations as presented by the current pandemic as well as for endangered parenting behavior. The ability to cope with stressful situations seems to have a significant influence on the context of a possible transgenerational transmission of CM experiences. Our results underline the need to consider the unique needs of families with children and to support them with a view on how to overcome the current crisis.

## Abbreviations

(CM): Childhood maltreatment
(CTQ): Childhood Trauma Questionnaire
(CSA): Experiences of child sexual abuse
